# The effects of a single session of spinal manipulation on strength and cortical drive in athletes

**DOI:** 10.1007/s00421-018-3799-x

**Published:** 2018-01-11

**Authors:** Thomas Lykke Christiansen, Imran Khan Niazi, Kelly Holt, Rasmus Wiberg Nedergaard, Jens Duehr, Kathryn Allen, Paul Marshall, Kemal S. Türker, Jan Hartvigsen, Heidi Haavik

**Affiliations:** 1 Department of Sports Science and Clinical Biomechanics, University of Southern Denmark, Odense, Denmark; 2 Center for Chiropractic Research, New Zealand College of Chiropractic, 6 Harrison Road, Mount Wellington, Auckland, New Zealand; 3 School of Science and Health, Western Sydney University, Sydney, Australia; 4 School of Medicine, Koç University, Istanbul, Turkey; 5 Nordic Institute of Chiropractic and Clinical Biomechanics, Odense, Denmark; 6 SMI, Department of Health Science and Technology, Aalborg University, Aalborg, Denmark

**Keywords:** Spinal manipulation, Athletic performance, Taekwondo, V-wave, H-reflex, Maximum voluntary contraction force, MVC, Fatigue

## Abstract

**Purpose:**

The primary purpose of this study was to investigate whether a single session of spinal manipulation (SM) increases strength and cortical drive in the lower limb (soleus muscle) of elite Taekwondo athletes.

**Methods:**

Soleus-evoked V-waves, H-reflex and maximum voluntary contraction (MVC) of the plantar flexors were recorded from 11 elite Taekwondo athletes using a randomized controlled crossover design. Interventions were either SM or passive movement control. Outcomes were assessed at pre-intervention and at three post-intervention time periods (immediate post, post 30 min and post 60 min). A multifactorial repeated measures ANOVA was conducted to assess within and between group differences. Time and session were used as factors. A post hoc analysis was carried out, when an interactive effect was present. Significance was set at *p* ≤ 0.05.

**Results:**

SM increased MVC force [*F*(3,30) = 5.95, *p* < 0.01], and V-waves [*F*(3,30) = 4.25, *p* = 0.01] over time compared to the control intervention. Between group differences were significant for all time periods (*p* < 0.05) except for the post60 force measurements (*p* = 0.07).

**Conclusion:**

A single session of SM increased muscle strength and corticospinal excitability to ankle plantar flexor muscles in elite Taekwondo athletes. The increased MVC force lasted for 30 min and the corticospinal excitability increase persisted for at least 60 min.

## Introduction

Athletic performance is influenced by a complex variety of physiological factors, such as neuromuscular coordination, muscle strength and endurance (Brutsaert and Parra 2006). Neuromuscular fatigue, reduced muscle strength and reduced power are all detrimental to athletic performance (Armstrong and McManus 2010; McManus and Armstrong 2011; Harries et al. 2012; Kockum and Heijne 2015) and the occurrence of these factors increases the risk of sports-related injuries (Marshall et al. 2014). These physiological factors are mediated by neuromuscular mechanisms and can be investigated by measuring maximum voluntary contraction (MVC) and spinal reflex responses such as the H-reflex, the M-wave and the V-wave (Milner-Brown and Lee 1975; Sale et al. 1983a; Aagaard et al. 2002; Tucker et al. 2005; Del Balso and Cafarelli 2007; Holtermann et al. 2007; Gondin et al. 2006; Vila-Cha et al. 2012; Marshall et al. 2014; Niazi et al. 2015). Improvement of neural adaptations, such as the H-reflex and the V-wave, contributes to enhanced motor performance (Pérot et al. 1991; Nordlund Ekblom 2010). By optimizing these physiological factors athletic performance may improve.

Previous studies have shown an increased excitability of the H-reflex associated with increased motoneuron (MN) excitability and enhanced resistance to fatigue, following strength training (Aagaard et al. 2002) and endurance training (Vila-Cha et al. 2012). A variety of studies have shown an increased amplitude of the V-wave associated with increased descending neural drive accompanied by increased MVC force and MN excitability following strength training (Milner-Brown and Lee 1975; Sale et al. 1983a, b; Aagaard et al. 2002; Nordlund Ekblom 2010; Vila-Cha et al. 2012). Similar neural adaptations have been found following spinal manipulation (SM) by Niazi et al. (2015), suggesting SM may have a similar neural plastic effect to what occurs with strength training as reported by Vila-Cha et al. (2012). It is, however, unknown whether such neural adaptations following SM also occur in a sports population.

SM is considered a safe and effective manual therapy for improving musculoskeletal conditions (Bronfort et al. 2010, 2012), such as acute and chronic low back pain, acute and chronic neck pain, shoulder pain and dysfunction, hip osteoarthritis, knee osteoarthritis, patellofemoral pain syndrome, plantar fasciitis, migrain headache and cervicogenic headache (Bronfort et al. 2010). Furthermore, SM may be cost-effective relative to other interventions used for these conditions (Tsertsvadze et al. 2014). In addition, there is evidence that SM alters a range of neurophysiological functions such as muscle reflexes and spinal pathways (Herzog et al. 1999; Niazi et al. 2015), neuromuscular fatigue (Niazi et al. 2015), cognitive processing (Kelly et al. 2000), reaction time (Lersa et al. 2005), cortical drive to the muscle (Niazi et al. 2015) and cortical somatosensory processing and sensorimotor integration (Haavik Taylor and Murphy 2007). SM has been reported to result in short-term increases in muscle strength (Hillermann et al. 2006; Botelho and Andrade 2012; Niazi et al. 2015). All of these neurophysiological functions are known to be crucial to athletic performance (Brutsaert and Parra 2006; Armstrong and McManus 2010; McManus and Armstrong 2011; Harries et al. 2012; Marshall et al. 2014; Kockum and Heijne 2015).

Previous studies have shown that although the H-reflex and V-waves are affected by common neural mechanisms, recording them both can differentiate between altered presynaptic inhibition and MN excitability (measured with the H-reflex) (Brooke et al. 1995; Pierrot-Deseilligny and Mazevet 2000; Hultborn 2006; Nordlund Ekblom 2010) and changes in supraspinal input to the MN pool (measured with the V-wave) (Sale et al. 1983a; Aagaard et al. 2002; Gondin 2006; Vila-Cha et al. 2012). The V-wave response is considered as an index of the cortical neural drive addressed to spinal alpha-motorneurons by some (Grosprêtre and Martin 2014). However, this is not yet universally accepted. Regardless, combining these measures may provide a better understanding of the changes that occur in the cortico spinal tract of an athletic (Taekwondo) population with SM. No previous study has investigated the effects of SM on these neurophysiological factors within an athletic population. Therefore, the purpose of this study was to evaluate the effects of a single session of SM on MVC force and neural reflex excitability (H-reflex, M-wave and V-wave) in athletes. We hypothesize that a single session of spinal manipulation (SM) will increase the maximal voluntary contraction (MVC) and that changes in corticospinal excitability to motor neurons (V-wave) will be greater than any change in H-reflex responses. We further hypothesize that the single-session SM will not affect the size of the M-wave responses.

## Methods

### Subjects

Twelve elite-level Taekwondo athletes with subclinical spinal pain (i.e. intermittent low-grade spinal pain, ache or tension) from the Auckland area of New Zealand participated in this study. One subject dropped out of the trial due to lack of interest, so data collection was completed on 11 subjects. All subjects gave their written informed consent. The study was approved by the Southern Health and Disability Ethics Committee, Auckland (15/STH/218/AM01), and this study was conducted according to the Declaration of Helsinki. The study was registered with the Australian New Zealand Clinical Trials Registry (ANZCTRN 12616000089437).

All subjects were required to be aged 17–50, be English speaking, have represented their country at the Taekwondo World Cup or World Championship during the previous 12 months and be actively engaged in resistance training at least twice per week on average over the previous 6 weeks. This is to be considered as an elite Taekwondo athlete in this study. Subjects were excluded if they had any absolute contraindications to SM (i.e. malignant cancer, metabolic disorders, inflammatory or infectious arthropathies), previously suffered from significant adverse reactions to SM (i.e. alleged disc herniation, treatment-induced fracture, organ injuries or vascular issues), had a recent history of trauma or were currently undergoing treatment elsewhere at the time of their inclusion in the study.

### Design

**Fig. 1:**
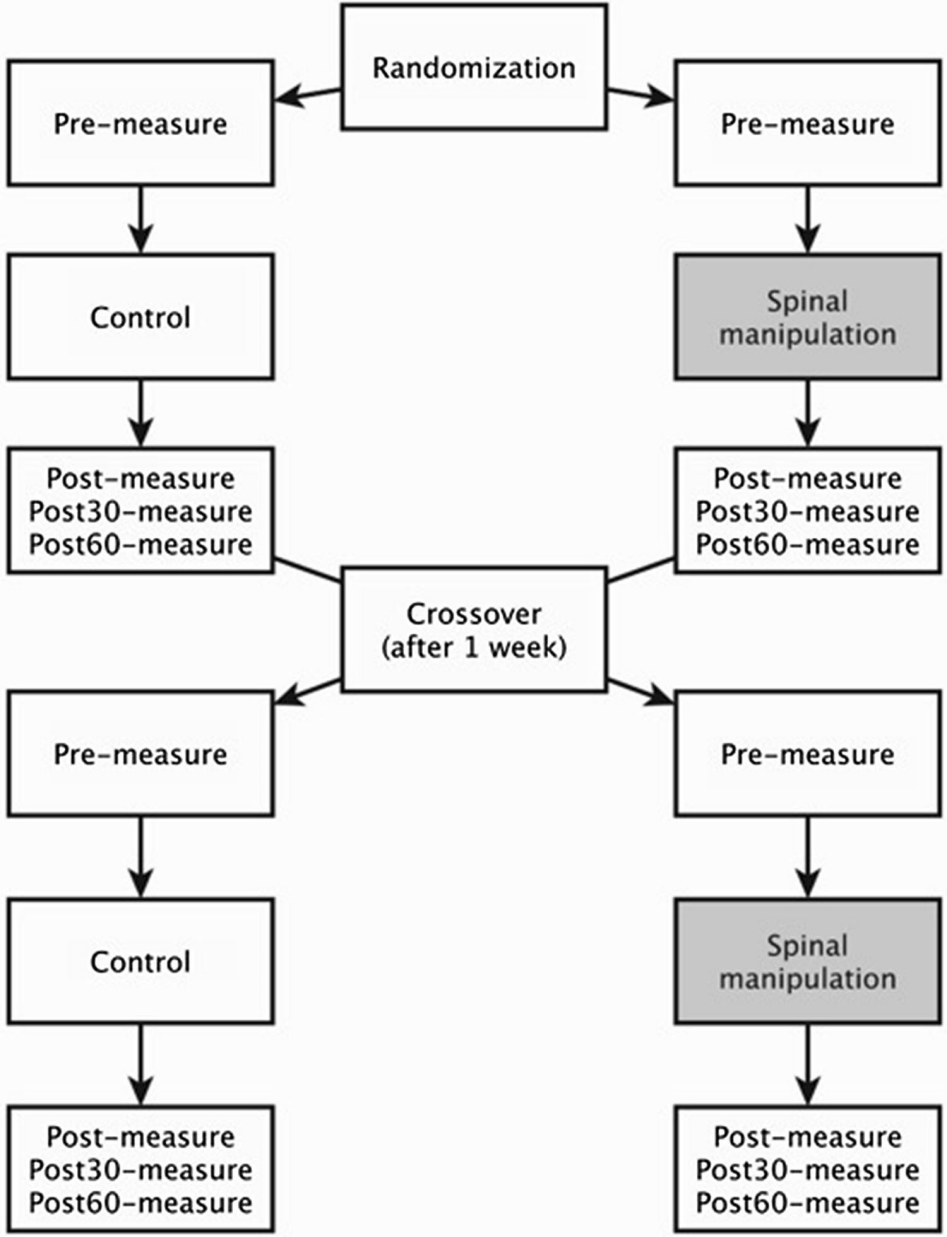
Flowchart illustrating the flow of subjects through the study

This study was a within-subject randomized controlled crossover trial with 1 week between sessions. The design used was a repeated measures design in which each subject received one intervention session, i.e. SM, and one control session, i.e. passive movements of head and spine. The subjects were randomly assigned to receive either SM or control intervention first and would then receive the alternate intervention 1 week later (see Fig. 1). The chiropractor providing the SM recorded a log of all SMs performed. The data collection was carried out at the Centre for Chiropractic Research at the New Zealand College of Chiropractic in Auckland, New Zealand.

### Sample size

Sample size calculations were based on detecting a difference in MVC’s between the control and intervention session and predicted effect sizes were based on changes observed in a previous study that investigated similar neurophysiological changes before and after SM (Niazi et al. 2015). If the true difference in MVC between the SM intervention and the control session had an effect size of 0.5, 11 subjects were needed to be able to reject the null hypothesis that the population means of the experimental and control groups are equal with probability (power) 0.9. To allow for drop out during the trial and relative uncertainty relating to power outcomes, we aimed to enroll 15 subjects in the trial.

### Randomization and blinding

Subjects were randomized using a computer-generated random number table, to first receive either SM intervention or control intervention. Chiropractors and subjects were not blinded to group allocation, as this would be difficult due to the SM intervention (Hancock et al. 2006; Rosner 2012). However, outcome-assessors and bioengineering data-analysts remained blinded to group allocation throughout the study period. Data file names were coded and sent to an independent data analyst to make sure this analyst was unaware of allocation.

### Interventions

#### Spinal manipulation

A licensed chiropractor assessed the function of the entire spine and both sacroiliac joints for segmental dysfunction (also known as vertebral subluxations by some chiropractors) and performed SM where he found it was indicated. The clinical indicators for segmental dysfunction include joint tenderness, restricted intersegmental range of motion, asymmetric intervertebral muscle tension, and abnormal or blocked joint play and end-feel of a joint. These biomechanical characteristics are used by chiropractors and other practitioners of manual therapy as clinical indicators of spinal dysfunction (Kockum and Heijne 2015). Subjectively observed restricted intersegmental range of motion and tenderness to touch are the most reliable clinical indicators of segmental dysfunction (Kockum and Heijne 2015), so these had to be present for SM to be performed. The SM performed in this study was high-velocity, low-amplitude (HVLA) joint manipulation, which is a standard SM technique used by chiropractors. The HVLA technique has previously been used in studies investigating the neurophysiological effects of SM (Haavik and Murphy 2012; Niazi et al. 2015).

#### Control session

During the control session, the head and spine of the subject were moved passively and actively, similar to the SM intervention but without the HVLA impulse. Loading a joint, as done prior to SM, is known to alter the paraspinal proprioceptive firing in anesthetized cats (Pickar and Wheeler 2001). Therefore to avoid this, the movement was ended prior to end range of motion when moving the subjects passively. The control session was intended to act as a physiological control for any possible changes occurring due to the time it took for the SM intervention, as well as any potential changes due to passive and active movement of the musculoskeletal system, which are also involved in preparing the subject for SM. Finally, the control session was also to act as a control for the contractions and stimulations necessary to obtain the study measures since, for example, it is well known that repeated maximum voluntary contractions lead to fatigue (Niazi et al. 2015).

### Setup

#### Subject preparation

The surface electromyography (sEMG) recording electrodes (20 mm Blue Sensor Ag–AgCl, AMBU A/S, Denmark) were placed on the innervation zones on the lateral aspect of the belly of the right soleus muscle (SOL) (i.e. about 5 cm below the gastrocnemius–Achilles tendon junction). To obtain low impedance (i.e. < 5 kΩ), relevant areas on the lower leg were shaved and dead skin cells removed using sandpaper and skin prepping gel. Alcohol swabs were used for cleaning the skin prior to attaching the bipolar sEMG recording electrodes (i.e. Ag/AgCl ECG conductive adhesive electrodes).

The H-reflex can be altered by posture (Schieppati 1987), so all subjects were comfortably positioned lying face down on a massage table and the right foot was placed in an immobile footplate. Dorsiflexion of the ankle is known to have an inhibitory effect on the MN pool of the SOL (Burke et al. 1984), so the foot was positioned in a neutral position. The subject’s arms were placed in a relaxed position alongside their torso and the subjects were told to focus on the task and not use their upper body or in any way alter their posture during the assessment.

#### Surface EMG

Using an electromyograph, it is possible to measure the electrical potentials generated by muscle cells stimulated by MNs, which are either cortically or electrically initiated. Like in previous studies (Brinkworth et al. 2007; Niazi et al. 2015), the bipolar sEMG recordings in this study were band-pass filtered (20–500 Hz) and amplified in a custom-made EMG amplifier with a builtin stimulus artefact suppressor and were recorded with a custom-designed computer program using CED (Power1401 mk 2) Data Acquisition Board at a sampling rate of 2 kHz. A grounding electrode was placed on the subjects’ tibial bone. Neurostimulating electrodes (Pals Platinum) evoked the H-, M- and V-wave of the SOL.

### Data recording of variables

Assessment of the outcome variables was made pre, immediately post, post30 and post60 SM intervention and control session. The following outcome variables were evaluated in the SOL, by electrically stimulating the tibial nerve.

#### Maximum voluntary contraction (MVC)

MVC force of the subjects (i.e. maximum isometric plantar flexion force), was measured using an isometric strain gauge (Model MLP100 transducer Techniques Tennecula, California, USA) mounted on a custom-built immobile footplate. The subjects initially performed three MVCs of the plantar flexor muscles of 5 s duration each, separated by “2-min rest” intervals, to optimize each MVC-recording. To maximize motivation during the procedure, subjects were verbally encouraged by the investigators to produce maximum force (Gandevia 2001). The strongest contraction, measured in absolute force, from each session, was used to compute the submaximal target contraction levels (i.e. 10% of MVC) for H- and M-recruitment curve recordings.

#### Maximum direct motor response (*M*_max_)

Following the three MVCs, the stimulation intensity needed to obtain *M*_max_ was determined by progressively increasing the stimulus intensity in 5 mA increments. This was done while assessing the size of the M-wave with visual feedback on the computer, to determine when the M-wave reached its maximum. Three stimulations were recorded at each current intensity, and the new peak-to-peak amplitude of the M-wave was compared with the preceding peak-to-peak amplitude. Once the peak-to-peak M-wave recordings reached a plateau, the M-wave was used for the respective normalization of either the H-reflex or the V-wave.

#### H- and M-recruitment curves

Following the M-wave calibration, the subjects performed a low-level tonic contraction of the Triceps Surae (i.e. 10% of MVC) while the M-wave and H-reflex of the SOL were elicited. The small contraction was necessary to maintain a steady level of MN excitability and minimize postsynaptic effects during the recordings of these dependent measures (Knikou 2008). To ensure the subjects were able to contract 10% of their MVC, they were provided with online feedback of their muscle contraction level, which was displayed by a moving bar on a clearly visible computer monitor.

Eighty stimuli in total were given within each assessment of the H-reflex and M-wave (i.e. pre, post, post30 and post60). The stimuli were triggered by a computer and delivered by a Digitimer constant current stimulator (model DS7A) with a square pulse of 1 ms duration in intervals of 0.5 s. The stimuli were separated in 16 stimuli intensities with five stimuli given within each intensity in random order (Brinkworth et al. 2007). The signals were equally separated on a logarithmic scale, showing the normal distribution of the H-reflex recruitment curve, and the M-wave as a sigmoid function.

#### V-wave and MVC

The change in absolute MVC force was obtained during V-wave measurements. Within each assessment, the subject performed five MVCs of 10 s duration separated by “2-min rest”-intervals. The rest-intervals were intended to optimize each MVC-recording which was used in the analysis of MVC force and fatigue. During these contractions, five supramaximal stimuli (110% of the current needed to evoke *M*_max_; 1 ms square pulse) were applied to the tibial nerve. The five supramaximal stimuli elicited the V-wave.

### Data analysis

#### Curve fitting

The size of the M-wave and H-reflex was calculated from the peak-to-peak amplitude of the averaged values of each stimulus intensity, which were curve-fitted (Brinkworth et al. 2007), so that the size and location of the H-reflex in relation to the M-wave could be normalized (i.e. stimulus normalization). The curve fitting was required to correctly determine the size of the H-reflex and the V-wave at various intensities along the recruitment curve. However, the size of the H-reflex and V-wave reflects factors that cannot be predicted and fixed; precision of stimulus delivery, excitability of the entire H-reflex arch (i.e. MN excitability, responsiveness of the Ia synapse), placement of the electrodes, skin resistance and accuracy of the recording. Because of this, the size of the H-reflex and V-wave response will differ not only between subjects, but from trial to trial in the same subject. When the results of the M-wave, H-reflex and V-wave were measured and calculated, the respective data were shaped using a hyperbolic function for the M-wave and a Gaussian for the H-reflex and V-wave (Brinkworth et al. 2007).

#### Normalization

When the curves had been recorded and modelled, they were normalized to observe any alterations over time and between sessions. *M*_max_ recorded in the pre-assessment served as a normalization factor for the H-reflex, M- and V-wave in that particular session. Thus, the H-reflex and V-wave were normalized to the corresponding *M*_max_ so the *H*/*M*_max_ and *V*/*M*_max_ ratios were calculated for each subject. The M-wave is affected by contraction intensity (Pensini and Martin 2004), thus the *M*_max_ used for the respective normalizations was elicited accordingly with either the H-reflex or the V-wave. The stimulus normalization was required to reduce inter-subject variability and obtain true changes in the excitability of the H-reflex and V-wave pathway. Because the M-wave was normalized to the stimuli and thus fixed, any significant shift in the H-reflex and V-wave recruitment curves would indicate a true change in the excitability of the pathway independent to alterations in the connection between electrode and nerve (Brinkworth et al. 2007).

#### H-reflex and V-wave

Following curve fitting and normalization, the H-reflexes were superimposed and subsequently averaged for each subject’s results from each assessment in each session. Then, all pre- and post-measures from each session were averaged to analyse any given alterations between sessions and over time. In a similar procedure, the peak-to-peak amplitude of the V-wave was also curve-fitted, normalized, superimposed, subsequently averaged and analysed to compare the results between sessions and over time.

#### Statistical analysis

All Pre- to post-intervention changes were evaluated using two-way ANOVA’s with time (Pre, Post, Post30, Post60) and intervention (Spinal Manipulation and Control) as factors. Post hoc pairwise comparisons were carried out using Tukey’s HSD tests to identify the specific differences. Significance level was set at *p* ≤ 0.05 for all observations.

## Results

We aimed to enroll 15 subjects in this crossover study, but only 12 subjects (six female; age, 25 ± 20 years; height, 170 ± 10 cm; weight, 60 ± 10 kg) were able to be recruited in the time available and 1 subject was excluded, because he did not finish both sessions due to lack of interest.

### H-reflex

**Fig. 2:**
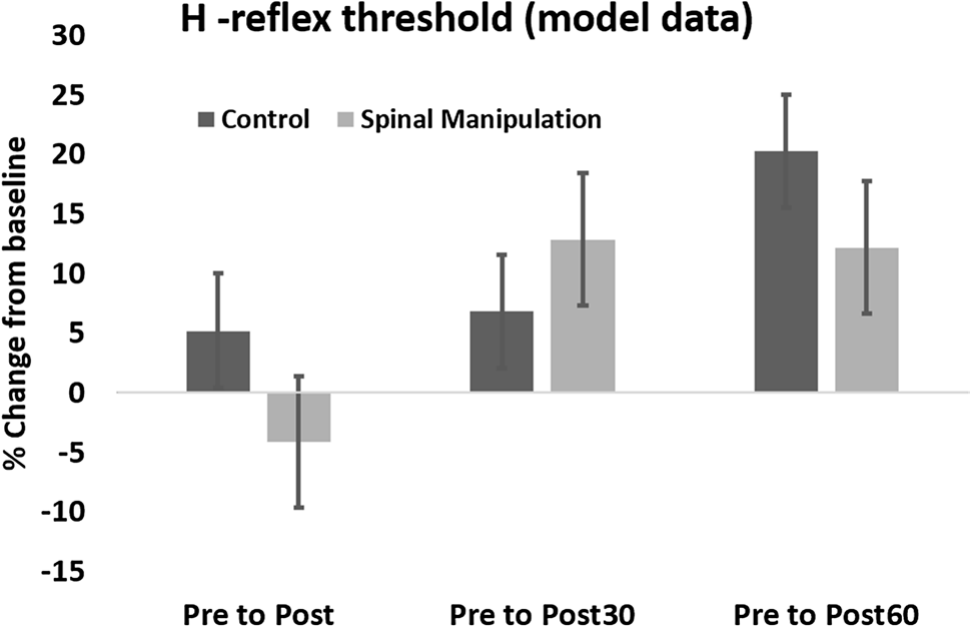
Relative changes in the H-reflex threshold of the model data in the SM intervention and control intervention. The dark columns show the H-reflex threshold in the control session and the bright columns shows the H-reflex threshold following SM. Error bar (std), *p* < 0.05

**Fig. 3:**
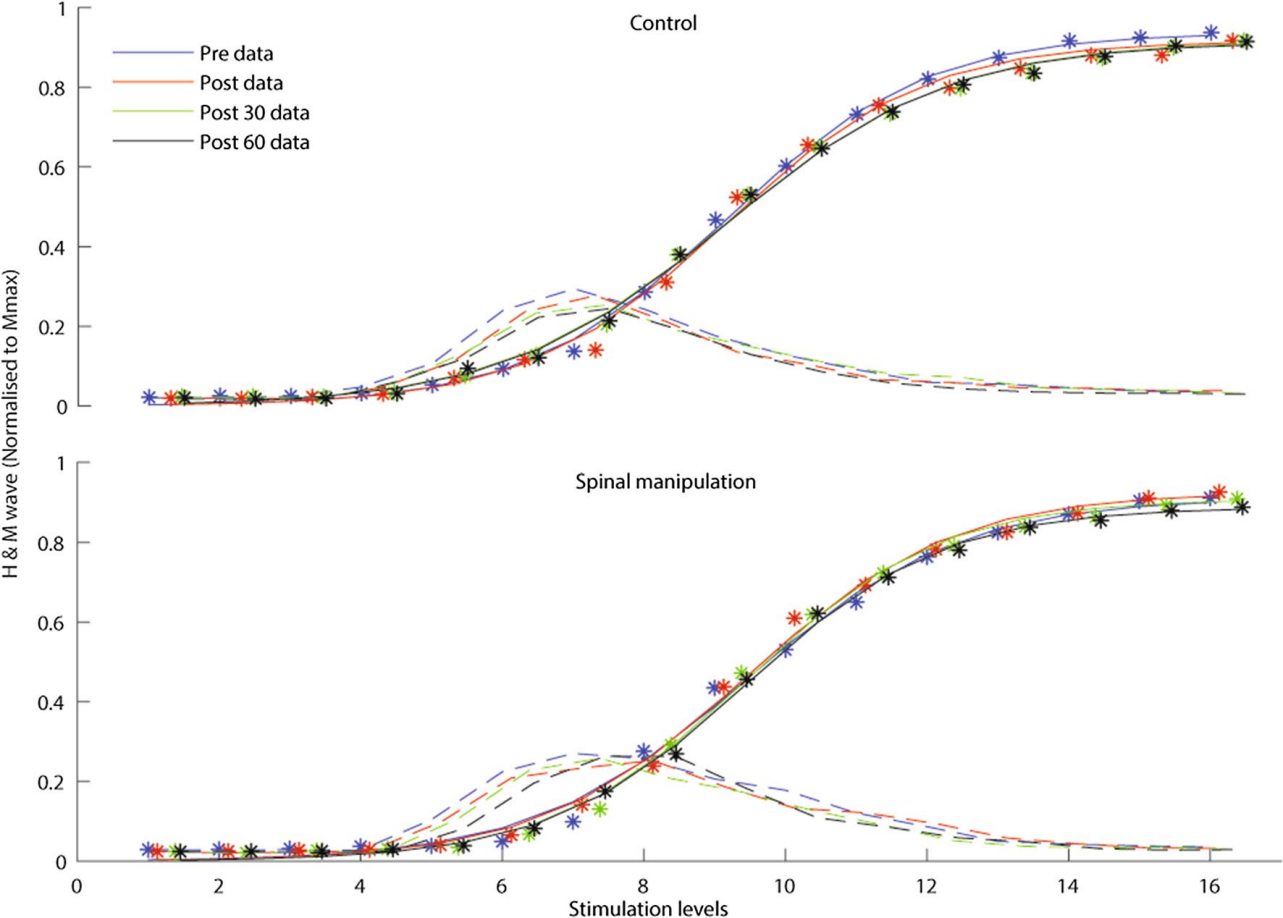
Recruitment curves of the H-reflex (-) and M-wave (●) illustrating changes in the amplitude of the H-reflex (H/Mmax ratio) in the control intervention (top) and SM intervention (bottom) of an average athlete, using the amplitude of the normalized responses against the stimulation levels. The colour of the time variables are presented in the top left corner. (Colour figure online)

The average relative changes from pre to post, post30 and post60 and their corresponding standard deviation from both sessions are presented in Table 1 and illustrated in Fig. 2. No significant difference [F (3,30) = 0.331 *p* < 0.80] was found in H-reflex threshold in the model data between the control and SM interventions. Figure 3 represents the fitted M-waves so that pre and post-M-waves curves could be superimposed on top of each other to allow any genuine changes in the H-reflex curve to be highlighted.

**Table 1:** Relative changes (∆) in the H-reflex threshold, V-wave amplitude and maximum contraction force in the control session and SM intervention and thier corresponding standard deviations (std)

	Pre to post	Pre to post30	Pre to post60
H-reflex			
Control			
∆ [%]	5.21	6.83	20.25
std [%]	52.37	39.26	35.54
SM			
∆ [%]	− 4.12	12.85	12.17
std [%]	40.64	44.00	66.32
*p* value	0.34	0.38	0.37
V-wave			
Control			
∆ [%]	− 11.73	− 21.34	− 21.97
std [%]	17.66	21.43	23.13
SM			
∆ [%]	33.72	40.57	46.20
std [%]	46.29	48.48	57.86
*p* value	0.03	< 0.01	< 0.01
Force			
Control			
∆ [%]	− 3.51	− 10.16	− 8.15
std [%]	9.30	11.06	12.15
SM			
∆ [%]	7.58	3.52	1.59
std [%]	7.37	9.50	7.42
*p* value	< 0.01	< 0.01	0.07

### V-wave

There was a significant difference in V-wave amplitude between the SM intervention and control intervention over time [*F*(3,30) = 4.25, *p* < 0.01]. Between intervention differences were also significant at each time period (*p* < 0.01–0.03). Following the SM intervention, the V-wave amplitudes increased significantly at all time points compared to baseline (*p* < 0.02–0.03).

**Fig. 4:**
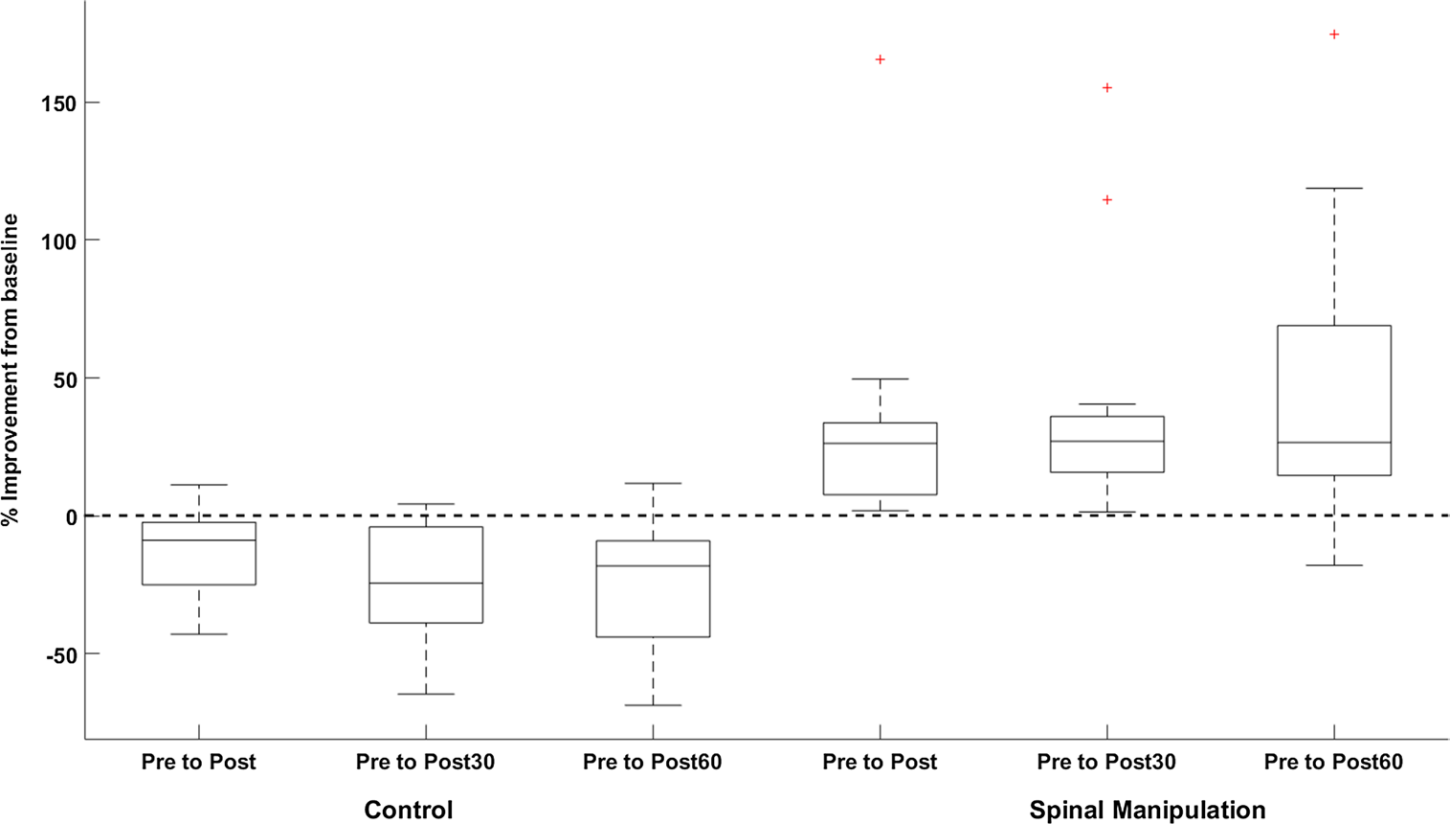
Relative changes in the V-wave amplitude (*V*/*M*_max_ ratio) of the SM intervention and control intervention. The first three columns show a decline in the V-wave in the control session and the last three columns show an improvement in the V-wave following SM. Error bar (std), *p* < 0.05

**Fig. 5:**
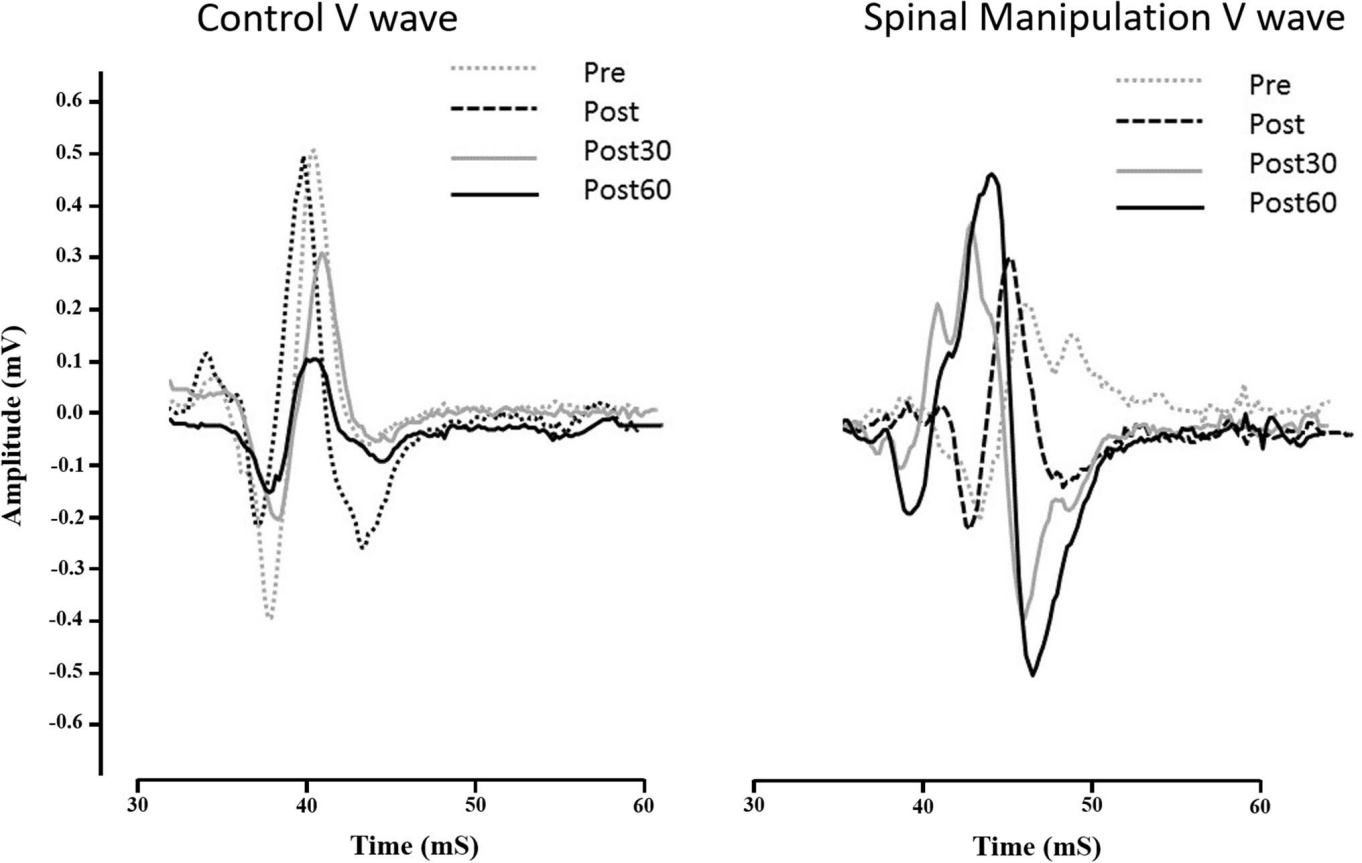
Change in the V-wave for a typical subject (*N* = 1). The graph illustrates average sEMG traces and the size of the V-wave before and after SM intervention and control intervention

Following the control intervention, V-wave amplitudes decreased at all time points compared to baseline. The immediate post-V-wave measurement decrease was not significantly following the control intervention (*p* < 0.2), but the post30 (*p* < 0.04) and post60 (*p* < 0.02) decreases were significant. Baseline differences between interventions were not significant (*p* < 0.1). The V-wave results are presented in Table 1 and illustrated in Figs. 4 and 5.

### Force

**Fig. 6:**
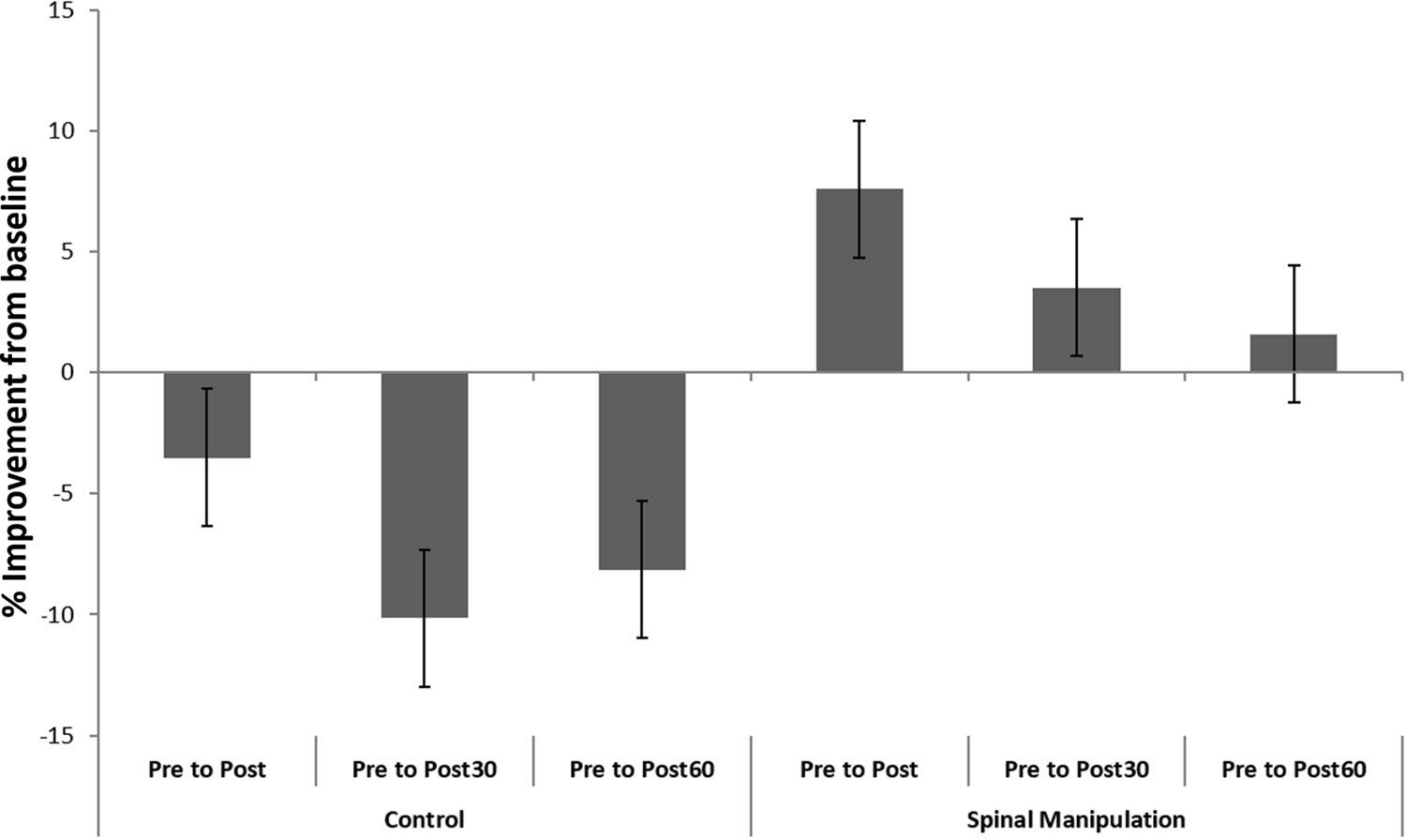
Relative changes of force measures in the control intervention and SM intervention. The first three columns show a decline in force following control session and the last three columns show an improvement in force following SM. Error bar (std), *p* < 0.05

There was a significant difference in MVC force between the SM intervention and control intervention over time [*F*(3,30) = 5.95, *p* < 0.01]. Between group differences were also significant at the immediate-post (*p* < 0.01) and post30 (*p* < 0.01) recordings, but they were no longer significant (*p* < 0.07) at the post60 recording. The MVC force increased significantly (*p* < 0.01) immediately post SM, but the increase in force were no longer significant at post30 (*p* < 0.3) and post60 (*p* < 0.6) recordings following the SM intervention. Following the control intervention MVC force decreased at all time points compared to baseline. The immediate post control MVC force decrease was not significant (*p* < 0.3), but the post30 (*p* < 0.01) and post60 (*p* < 0.02) decreases were significant following the control intervention. Baseline differences between groups were not significant (*p* < 0.2). The MVC force results are presented in Table 1 and illustrated in Fig. 6.

## Discussion

The main finding in this study was that maximum plantar flexion force and corticospinal excitability to the plantar flexors (i.e. V-wave) increased following SM but not in the control group. MVC force decreased over time in the control intervention but increased following the SM intervention, possibly as a result of increased cortical drive to the muscle. This is to our knowledge the first study demonstrating such neurophysiological alterations following SM to be investigated within an elite athletic population.

No H-reflex parameters changed significantly in this study. This supports previous research suggesting that changes in strength may be caused by supraspinal changes and not changes in the H-reflex (Alkjaer et al. 2013; Un et al. 2013). Niazi et al. found an increased excitability of low-threshold MNs in the H-reflex following SM within a population of healthy young males (Niazi et al. 2015), whereas Suter et al. found a decreased MN excitability in the H-reflex following SM within a low back pain population (Suter et al. 2005). Thus indicating, that the effects of SM on the H-reflex pathway may depend on the population.

The increased V-wave amplitudes observed in the current study possibly reflect an increased cortical drive in the cortico-spinal pathways and corresponding increased excitability of the MNs following SM (Aagaard et al. 2002; Pensini and Martin 2004; Vila-Cha et al. 2012; Niazi et al. 2015). Un et al. found differences in the cortical drive in volleyball athletes competing at different levels, and argued that elite players had increased cortical drive correlating to their biomechanical performance (Un et al. 2013). The absence of change in the H-reflex in the presence of the increased MVC along with increased V-waves suggests that its possible that the change post manipulation occurred at supraspinal centres involving cortical neural drive. Grosprete and Martin (2014) argue the V-waves represent cortical drive. The absence of change in the H-reflex alone suggests that the spinal motor neurons and the excitability of the spindle primary afferent synapses on the spinal motor neurons did not change as a result of SM. However, as the V-wave is not universally accepted as a measure of cortical drive this should still be interpreted with caution. It is clear that spinal manipulation alters corticospinal excitability, however, further research is still needed to dissociate between supraspinal and cortical influences.

Increases in V-wave amplitudes have also been shown following resistance training (Milner-Brown and Lee 1975; Sale et al. 1983a, b; Aagaard et al. 2002; Gondin 2006; Del Balso and Cafarelli 2007; Holtermann et al. 2007; Vila-Cha et al. 2012; Alkjaer et al. 2013). Alkjaer et al. found that 4 weeks of intensive drop jump training increased the athletes’ maximum jumping height with increased V-wave amplitude and without any significant increases in muscle strength or rate of force development, and argued therefore that the improved jumping performance was caused by central neural factors (Alkjaer et al. 2013). Sale et al. (1983a, b) and Milner-Brown and Lee (1975) found that weight-lifters had an elevated V-wave amplitude (Milner-Brown and Lee 1975; Sale et al. 1983b). Vila-Cha et al. (2012) and Aagaard et al. (2002) reported that strength training improved the V-wave peak-to-peak amplitude (measured as *V*/*M*_max_ ratio) (Aagaard et al. 2002; Vila-Cha et al. 2012). The findings of these studies suggest that neural adaptations related to cortical drive may occur in the initial phases of resistance training and then level off after 3 weeks. In the present study, increased V-wave amplitudes had occured following SM in 11 elite Taekwondo athletes who were actively engaged in resistance training at least twice per week on average over the previous 6 weeks. Thus, the increased V-wave following SM indicates a potential further improvement in athletic performance in highly trained athletes over and beyond resistance training.

In the current study, the increased V-wave amplitude was associated with improvements in maximum plantar flexion force following SM. A small number of studies have investigated changes in muscle strength following SM, in both athletes and non-athletes, with conflicting results (Suter et al. 1999; Hillermann et al. 2006; Botelho and Andrade 2012; Humphries et al. 2013; Niazi et al. 2015). Niazi et al. (2015) reported increases in muscle strength in subjects with subclinical pain following SM (Niazi et al. 2015), and Chilibeck et al. (2011) reported that in subjects with imbalances in lower limb muscle strength, SM resulted in increased muscle strength of hip abductors in their weak leg (Chilibeck et al. 2011). In athletes, Botelho (2012) reported increases in grip strength in national level judo athletes following SM (Botelho and Andrade 2012), but Humphries et al. (2013) found no changes in handgrip strength in asymptomatic basketball players following SM (Humphries et al. 2013).

In the current study, assumptions can be made about the effects of SM on neuromuscular fatigue based on changes in MVC force in the two interventions. After the control intervention MVC force decreased, suggesting that subjects were fatiguing, but no fatigue was observed following the SM intervention. Neuromuscular fatigue is known to decrease muscle strength and power, and is further a primary contributory factor for musculoskeletal injuries in exercise performance (Marshall et al. 2014), making it detrimental to athletic performance.

This study supports a growing body of research that suggests chiropractic spinal manipulation’s main effect is neuroplastic in nature and affects corticospinal excitability (Haavik-Taylor and Murphy 2007; Haavik and Murphy 2012; Lelic et al. 2016; Haavik et al. 2017). Changes in both cerebellum (Daligadu et al. 2013) and prefrontal cortex (Lelic et al. 2016) function have been implicated post-spinal manipulation in previous research studies. The presence of mild, recurrent spinal dysfunction has been shown to be associated with maladaptive neural plastic changes, such as alterations in elbow joint position sense (Haavik and Murphy 2011), mental rotation ability (Baarbé et al. 2016), and even multisensory integration (Farid et al. 2017). Furthermore, spinal manipulation of dysfunctional spinal segments have been shown to impact somatosensory processing, sensorimotor integration and motor control (Haavik-Taylor and Murphy 2007; Taylor and Murphy 2008, 2010a, b; Haavik and Murphy 2011, 2012; Haavik et al. 2017). Thus, it is likely that mild spinal dysfunction alters CNS function that impacts motor control, and that this may further be impacted during motor training. Recently, Andrew et al. (Andrew et al. 2017) showed that participants with mild, recurrent neck pain displayed different CNS effects following an upper limb learning task compared to those with no history of any neck dysfunction, despite all subjects being pain free on the day of testing. The current study findings suggest chiropractic care may be of benefit even for subjects without pain to improve muscle performance. However, further research is still needed to elucidate how such neurophysiological changes may impact sports performance, strength training and/or other behavioural measures.

### Strengths and limitations

One limitation of the current study is the small sample size that was included. The targeted sample size of 15 was based on changes observed in a previous study conducted in subclincial pain subjects (Niazi et al. 2015). However, the effect size in the current study was smaller than this previous study. This should be expected as a population of elite athletes may have less room for improvement in strength and fatigue compared to subjects who are not highly trained. Compounding this issue only 11 subjects successfully completed the study. This may mean that some or the within and between group differences that were not significant may have been due to the study being underpowered. For example, between group post60 strength changes were not significant (*p* < 0.07). This may mean that changes in strength persist for less than 60 min after SM, or it may mean that a type II error occurred, so this conclusion may be incorrect. The same may be true for the H-reflex measures. This study can, therefore, not exclude the possibility that small H-reflex changes may occur following SM.

To test the hypothesis whether a single session of SM will change MVC and V wave, crossover design was used. One strength of this randomized controlled crossover trial is that the individual athlete acts as their own control. To reduce carry-over effects, the order of receiving SM was randomized, thereby equalizing any potential benefits from the previous assessment. No significant changes were observed in the baseline data analysis, suggesting no carry-over effect occured in this study.

MVC force is an objective measure, but the results can be misrepresentative as it depends on subject participation (Gandevia 2001). To avoid this bias, the subjects could have had a practice session a few days before the first real session. As this was not done it is possible such a training bias did occur (Oliveira et al. 2010), although this would have been equally distributed for both sessions as the order of session was randomized. These issues can be further explored in future studies using twitch interpolation techniques as well (Gandevia et al. 2013). Finally, due to the nature of the interventions no blinding of participants was attempted (Rosner 2012). This results in the possibility of placebo effects or performance bias.

### Clinical and research implications

Increased strength, reduced neuromuscular fatigue, and increased cortical drive are crucial factors to athletic performance, so by optimizing these physiological factors athletic performance may improve (Armstrong and McManus 2010; McManus and Armstrong 2011; Harries et al. 2012; Marshall et al. 2014; Kockum and Heijne 2015). Additionally, athletes have reported improvement in athletic performance (Brolinson et al. 2012; Nook et al. 2016) and reduction in pain (using VAS-score) following SM (Nook et al. 2016). Thus, emphasizing that SM could be used to enhance athletic performance, with the advantage of it being drug-free, safe and cost-effective (Bronfort et al. 2010, 2012; Tsertsvadze et al. 2014).

Future studies should investigate athletic performance in “real-life”-studies, while having the athletes execute suitable exercises related to a given sport. It would be relevant to investigate different athletic populations, especially various types of power and endurance athletes, as these are known to respond differently neurophysiologically (Vila-Cha et al. 2012). Moreover, it would be relevant to consider gender and age of the athletes, as physiological factors differ within these categories as well (Brutsaert and Parra 2006; Armstrong and McManus 2010; McManus and Armstrong 2011).

## Conclusion

A single session of SM of dysfunctional spinal and pelvic joints increased muscle strength and cortical drive to ankle plantar flexor muscles in elite Taekwondo athletes. The increased MVC force lasted for 30 min and the cortical drive increase persisted for at least 60 min. Further research is now required to determine whether the observed changes are important for athletic performance.

## Abbreviations

H-reflex: Hoffmann’s reflex
HVLA: High-velocity, low-amplitude
MVC: Maximum voluntary contraction
MN: Motoneuron
Mmax: Maximum direct motor response
SM: Spinal manipulation
sEMG: Surface electromyography
SOL: Soleus muscle
